# smsMap: mapping single molecule sequencing reads by locating the alignment starting positions

**DOI:** 10.1186/s12859-020-03698-w

**Published:** 2020-08-04

**Authors:** Ze-Gang Wei, Shao-Wu Zhang, Fei Liu

**Affiliations:** 1 Key Laboratory of Information Fusion Technology of Ministry of Education, School of Automation, Northwestern Polytechnical University, Xi’an, 710072 China; 2 Institute of Physics and Optoelectronics Technology, Baoji University of Arts and Sciences, Baoji, 721016 China

## Abstract

**Background:**

Single Molecule Sequencing (SMS) technology can produce longer reads with higher sequencing error rate. Mapping these reads to a reference genome is often the most fundamental and computing-intensive step for downstream analysis. Most existing mapping tools generally adopt the traditional seed-and-extend strategy, and the candidate aligned regions for each query read are selected either by counting the number of matched seeds or chaining a group of seeds. However, for all the existing mapping tools, the coverage ratio of the alignment region to the query read is lower, and the read alignment quality and efficiency need to be improved. Here, we introduce smsMap, a novel mapping tool that is specifically designed to map the long reads of SMS to a reference genome.

**Results:**

smsMap was evaluated with other existing seven SMS mapping tools (e.g., BLASR, minimap2, and BWA-MEM) on both simulated and real-life SMS datasets. The experimental results show that smsMap can efficiently achieve higher aligned read coverage ratio and has higher sensitivity that can align more sequences and bases to the reference genome. Additionally, smsMap is more robust to sequencing errors.

**Conclusions:**

smsMap is computationally efficient to align SMS reads, especially for the larger size of the reference genome (e.g., *H. sapiens* genome with over 3 billion base pairs). The source code of smsMap can be freely downloaded from https://github.com/NWPU-903PR/smsMap.

## Background

Technological breakthroughs in high-throughput sequencing (HTS) platforms have triggered a revolution in genomics [1–5], which enable scientists to obtain the full genomic sequence of many species by read alignment and de novo assembly [6–8]. Mapping (or aligning) the HTS reads from a sample to a reference genome is the most fundamental and computing-intensive step in genome resequencing studies [7, 8], which detects genome variations such as single nucleotide polymorphisms, large-scale structural variations (SVs) and count for different sequencing errors [9, 10]. All existing tools for mapping short reads, such as BLAST [11], BLAT [10], SOAP2 [12], Bowtie [13] and BWA [14], aim to find a “long” substring that would exactly match its mapping locus on the reference genome by using either Burrows-Wheeler Transform Full-text Minute-space (BWT-FM) index [15, 16], or substring hashing [17], or hybrid methods of combining FM index with hashing [18]. However, due to a higher number of errors which are primarily insertions and deletions rather than substitutions, the mapping methods created for short reads are not readily extended to long reads generated from the single molecule sequencing (SMS) technology [19].

Recently, a number of available methods (or tools) for mapping SMS long reads to the reference genome, such as BLASR [20], BWA-MEM [21], rHAT [22], GraphMap [23], LAMSA [24], minimap2 [25], NGMLR [26] and lordFAST [27], have been proposed. BLASR [20] is the first tool that is specially designed for mapping SMS reads. It first builds a BWT-FM index [15, 16] of the genome to search exact matches and then applies sparse dynamic programming (SDP) to generate rough alignments. The final detailed alignments are generated by dynamic programming. BWA-MEM [21] initially finds the alignment seeds and greedily chains these seeds, then extends the selected seeds to achieve the alignment. rHAT [22] utilizes the regional hash table (RHT) to find the highest possible candidate regions and then adopts an SDP-based approach to align the reads on the candidate regions. GraphMap [23] finds gapped space seeds that are clustered to obtain a coarse alignment, then uses a graph-based vertex-centric procedure to construct the alignment anchors, and chains these anchors, refining the chain to generate the final alignment. LAMSA [24] finds all approximate matches on the reference genome by using the GEM mapper [28], it then builds a direct acyclic graph (DAG) to generate alignment skeletons. Finally, LAMSA implements a specific split-alignment strategy to fill the gaps within the skeletons. minimap2 [25] collects minimizers [29] of the reference genome sequence for indexing them in a hash table and then finds matches to the reference by identifying the sets of co-linear seeds. Afterward, minimap2 applies dynamic programming in the unseeded regions to get the final alignment. NGMLR [26] first finds the linear mapping seeds, then performs a pairwise sequence alignment based on the Smith-Waterman algorithm. NGMLR lastly selects the set of linear alignments with the highest joint score as the final read alignment results. lordFAST [27] first builds an index from the reference genome then maps reads to the reference genome by extracting longest exact matches. It next selects candidate alignment regions, and finally gets the base-to-base alignment with dynamic programming.

All of the above mapping methods designed for SMS reads follow the canonical seed-and-extension paradigm [18, 30], that is, they find the maximal exact matches (seeds) and then extend the alignment to the non-seed fragments within the selected candidate regions in the query read and the reference genome. The major differences among them are the ways by which seeds and the candidate aligned region are selected.

The candidate aligned regions for each query read are selected either by counting the number of matched seeds (e.g., rHAT and lordFAST) or chaining a group of seeds that are co-linear or close to each other (e.g., BLASR, LAMSA, GraphMap, NGMLR, BWA-MEM, and minimap2). However, the candidate aligned region of each query read is always a part of the read and cannot completely cover the whole read length. Although the non-seed fragments are subsequently aligned with dynamic programming, they are still within the candidate regions. For the two ends of the query read, which are not covered by the candidate aligned region, all existing methods do not align them and directly output as the soft clipping. As a result, these methods designed for SMS reads usually focus on producing local mapping results for the query read, other than obtaining the whole end-to-end alignment, leading to low aligned coverage (aligned fraction of the read). The aligned coverage is the percentage of one query sequence aligned to a reference genome, which reflects the effectively aligned size of the query sequence. Many researchers consider the alignments with higher aligned coverage as the valid alignment results [31–34]. Additionally, alignments with higher aligned coverage mean that more aligned bases can be obtained, which is a key requirement for mapping tools and mapping-based analysis [23], as bases that cannot be mapped are unavailable for use in many downstream applications [35–37]. Therefore, the shortcoming (i.e., low aligned coverage) of current mapping methods highlights the need for a sensitive, efficient computational method with higher aligned coverage.

Herein, we proposed a new SMS sequence mapping method (called smsMap) that aims to get the end-to-end accurate alignment against the reference genome for a query read. smsMap mainly contains three steps. It first constructs the BWT-FM index for the reference genome, then finds the starting positions in the query read and reference genome, and lastly a column reduction banded alignment method is developed to obtain the detailed dynamic alignment results from the located starting positions to the two ends of the query read and the genome, which can cover the whole read length. The experiments on simulated and real-life PacBio datasets show that smsMap can achieve more aligned coverage than other mapping tools. Also, smsMap is more sensitive that can map more reads and bases onto the reference genome.

## Results

smsMap is implemented in C++ language with multithreading, and it can be run in both Linux and Windows systems. To evaluate the performance and efficiency of smsMap, we compared our smsMap with other seven state-of-art long read mapping tools, such as BLASR [20], BWA-MEM [21], GraphMap [23], minimap2 [25], NGMLR [26], rHAT [22] and lordFAST [27] on simulated datasets and real-life datasets. Because LAMSA [24] always appears a segmentation fault (core dumped) information, we did not compare with it. The real-life raw sequencing datasets, derived from *E. coli*, *A. thaliana*, *C. elegans* and *H. sapiens* (CHM1), were generated by PacBio sequencing platform. All methods were executed on an Ubuntu 16.04.5 server with 16 3.2-GHz Intel Xeon (E5-2667V4) processors and 128 GB of RAM. The parameters used for each mapping tools are given in Table S1.

For simulated sequence datasets, if a read is aligned to the correct genome and strand, and the aligned subsequence on the reference genome overlaps with the “true” mapping subsequence by at least *p* bases (here *p* = 0.9*L*(*r*_*τ*_)), we consider this read to be correctly mapped on the genome [27]. If a matched base locates within *T* bp (here *T* = 5) of the corresponding truth position on the genome [24, 27], we consider this base as a correct matched base. Thus, we use three measures including the fraction of correctly aligned reads (cFAR), the fraction of correctly aligned bases (cFAB), and the average coverage ratio of correctly aligned reads (cACR) to estimate the performance of mappers on simulated datasets, which are defined as the following percentages:

1 $$ \left\{\begin{array}{l} cFAR=\frac{N^c}{N}\kern0.5em \times \kern0.5em 100\%\\ {} cFAB=\sum \limits_{\tau =1}^{N^c}\frac{M_{\tau}^c}{M}\kern0.5em \times \kern0.5em 100\%\\ {} cACR=\frac{1}{N^c}\sum \limits_{\tau =1}^{N^c}\frac{M_{\tau}^c}{M_{\tau }}\times \kern0.5em 100\%\end{array}\right. $$

where *N*^*c*^ is the total number of correctly aligned reads, *N* is the total number of query reads, ${M}_{\tau}^c$ is the number of correct matched bases for read *r*_*τ*_, *M*_*τ*_ is the number of matched bases for read *r*_*τ*_, *M* is the total number of matched bases for all correctly aligned reads. An example of how to calculate cFAR, cFAB and cACR is presented in the supplementary file. Additionally, for simulated sequence datasets, base sensitivity and precision [27] are used to compare the performance of different mappers. Sensitivity is defined as the number of correct matched base divided by the total number of bases, precision is defined as the number of correct matched bases divided by the number of mapped bases.

Due to the true base pairing on the reference genome unknown for real-life read datasets, we use another three measures of the fraction of aligned reads (FAR), the fraction of aligned bases (FAB), and the average coverage ratio of aligned reads (ACR) to estimate the performance of mappers on real-life datasets, which are defined as the following percentages:

2 $$ \left\{\begin{array}{l} FAR=\frac{N^a}{N}\kern0.5em \times \kern0.5em 100\%\\ {} FAB=\sum \limits_{\tau =1}^{N^a}\frac{M_{\tau}^a}{M}\kern0.5em \times \kern0.5em 100\%\\ {} ACR=\frac{1}{N^a}\sum \limits_{\tau =1}^{N^a}\frac{M_{\tau}^a}{M_{\tau }}\times \kern0.5em 100\%\end{array}\right. $$

where *N*^*a*^ is the total number of aligned reads, *N* is the total number of query reads, ${M}_{\tau}^a$ is the number of matched bases for read *r*_*τ*_, *M*_*τ*_ is the base number of read *r*_*τ*_, *M* is the total base number for all query reads.

### Evaluation on simulated datasets

#### Simulation without structural variations

We first adopted the simulated datasets without structural variations (SVs) to evaluate the performance of our smsMap and other mapping tools. The *E. coli* MG1655 genome sequence (with the length of 4,614,652 bp) from NCBI (No. NC_000913.3) was downloaded and inputted to the NPBSS simulator [19] for generating the PacBio simulated reads with different error rates. As a result, 6 simulated datasets with 5, 10, 15, 20 25 and 30% error rates were generated. The sequencing depth and average read length are 50 and 10,000 bp, respectively. The error parameter settings of NPBSS can be found in Table S2. The reads number and total bases of each simulated dataset are listed in Table S3.

**Fig. 1:**
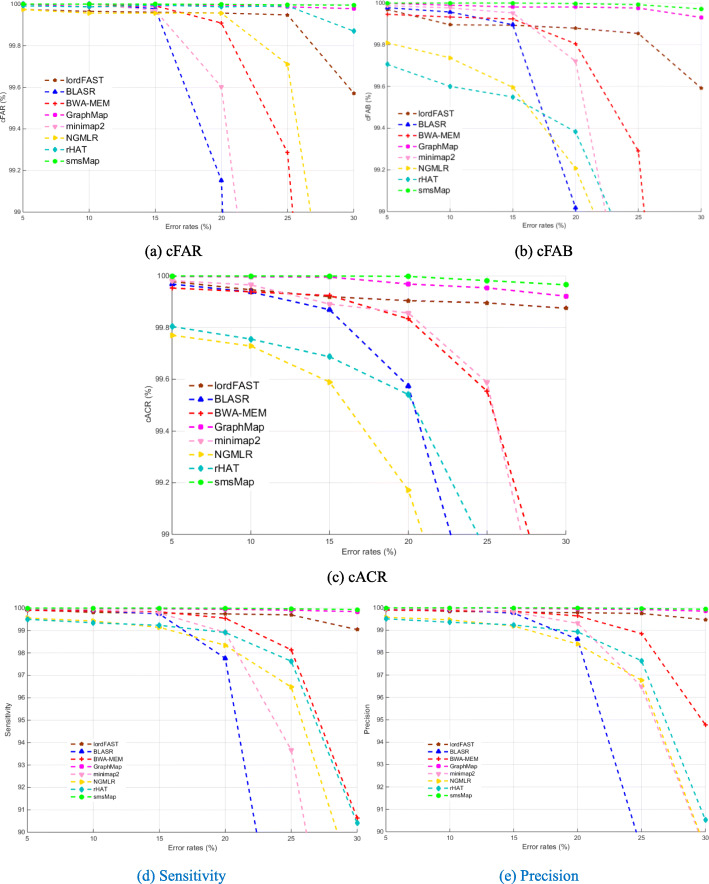
Mapping results of eight methods on the simulated datasets with different error rates

Figure 1 shows the cFAR, cFAB, cACR, sensitivity, and precision of smsMap, BLASR, BWA-MEM, GraphMap, minimap2, NGMLR, rHAT and lordFAST on the simulated datasets with different error rates (Table S4 gives the detail results of these methods). From Fig. 1, we can see that smsMap and GraphMap correctly mapped almost all reads and bases with different error rates (i.e., 5 to 30%) to the genome, while the cFAR, cFAB of other six mappers gradually decrease as the read error rates increase. smsMap, lordFAST, and GraphMap achieved higher cACR than the other five methods with different error rates, but our smsMap obtained little higher cACR than lordFAST and GraphMap, especially when the error rate is more than 20%. For the base sensitivity and precision in Fig. 1d and e, we can see that the sensitivity and precision of smsMap, minimap2, lordFAST, BLASR, and GraphMap are significantly higher than those of rHAT and NGMLR with error rate increases from 5 to 15%. For error rates ranging from 15 to 30%, smsMap and GraphMap achieved higher sensitivity and precision than other methods, and smsMap obtained a little higher sensitivity and precision than GraphMap. These results show that our smsMap are more robust to sequencing errors, and it can obtain better mapping quality for simulated datasets without SVs. Tables S5 lists the alignment scores for different methods with parameters: match = 2, mismatch = − 2, gap existence = − 2 and gap extension = − 2. It can be seen that the average scores are various among different methods.

Another simulated dataset of *H. sapiens* (CHM1) generated by NPBSS was also applied to test the performance of different methods. Table S6 shows the cFAR, cFAB, cACR, base sensitivity and precision of smsMap, BLASR, BWA-MEM, GraphMap, minimap2, NGMLR, rHAT and lordFAST on the simulated dataset of *H. sapiens* (CHM1). We can see that smsMap still achieved better mapping results in terms of cFAR, cFAB, cACR, sensitivity, and precision.

#### Simulation with structural variations

In order to estimate the capability of smsMap for mapping reads that span structural variations (SVs), we used another simulation dataset from chr1 of NA12878 with SVs. The simulation dataset with SVs was generated by inserting 7 SVs (i.e., 3 insertions, 3 deletions and 1 inversion) from DGV [38] into the reference chr1 and using the NPBSS simulator [19] at 20x coverage. Among the simulated reads, a total of 185 reads cover the SVs breakpoints. The detailed SVs and its breakpoints are listed in Table S7.

If the start and end alignment coordinates of a read in the genome cover the actual simulated breakpoints, we consider this read spanning SVs [27]. Here, we provide the aligned reads number of spanning SVs (#SVs) to evaluate the performance of different mapping tools. The results of our smsMap and other seven tools are listed in Table 1, from which we can see that our smsMap can map more reads with SVs on the genome than the other six tools, suggesting that our smsMap can also handle the SV-spanning reads.

**Table 1:** The number of aligned reads that span SVs breakpoints for different methods

	smsMap	lordFAST	BLASR	BWA-MEM	GraphMap	minimap2	NGMLR	rHAT
#SVs	126	113	30	100	105	81	73	79

### Evaluation of the real datasets

In addition to the simulated datasets, we also used four datasets (generating by PacBio RS II) of *E. coli*, *A. thaliana*, *C. elegans* and *H. sapiens* (CHM1) to further test the performance of our smsMap. The raw sequences, assembly genome, and statistics of these datasets can be found in Tables S8, S9, S10, respectively. Considering that rHAT always returns the segmentation fault (core dumped) information, we do not provide the results of rHAT in the real datasets.

Table 2 reports the mapping results of seven methods in four real datasets, and Table 3 lists ACR standard deviation (std). From Table 2, we can see that FAR of our smsMap is respectively 3.16–24.69%, 2.18–12.12%, 0.58–4.38%, and 3.75–9.86% higher than those of other six methods for *E. coli*, *A. thaliana*, *C. elegans* and *H. sapiens* datasets, and FAB is respectively 1.48–12.05%, 4.28–16.71%, 1.84–10.71%and 1.29–17.29% higher than those of other six methods for *E. coli*, *A. thaliana C. elegans* and *H. sapiens*. These results indicate that smsMap can map much more reads and bases to the genome for real datasets. FAR of our smsMap is respectively 0.42–6.41%, 0.16–7.48%, 0.21–6.32%, and 3.75–9.00% higher than those of other six methods for *E. coli*, *A. thaliana*, *C. elegans,* and *H. sapiens*, indicating that the aligned reads of smsMap can overlap more segments of the query reads. From Table 3, we can find that the ACR std. of smsMap is also significantly smaller than those of other six methods, indicating that smsMap can generate better mapping quality for real datasets. Additionally, one real dataset (i.e., *E. coli* UTI89) generated by MinION sequencer was used to evaluate the performance of seven methods for Oxford Nanopore sequencing data [23]. Table S11 reports the mapping results for *E. coli* UTI89 dataset, from which we can also observe that smsMap achieved higher FAR, FAB, and ACR than other methods, demonstrating that smsMap generates better mapping quality for Oxford Nanopore sequencing data.

**Table 2:** FAR(%), FAB(%), and ACR(%) of seven methods on four real datasets

	Datasets	smsMap	lordFAST	BLASR	BWA-MEM	GraphMap^a^	minimap2	NGLMR
FAR	*E. coli*	97.452	72.760	94.285	92.201	94.217	89.886	91.685
	*A. thaliana*	99.912	92.611	97.728	97.255	93.731	95.334	87.787
	*C. elegans*	99.018	94.630	98.430	98.333	96.975	97.540	95.011
	*H. sapiens*	99.182	90.280	97.921	96.651	–	94.762	90.722
FAB	*E. coli*	99.998	93.207	90.909	89.729	98.514	89.923	87.941
	*A. thaliana*	99.980	95.700	91.328	90.282	94.115	90.666	83.262
	*C. elegans*	99.623	93.564	93.602	91.688	97.783	93.206	88.904
	*H. sapiens*	99.956	93.923	91.588	90.321	–	92.349	85.219
ACR	*E. coli*	99.999	98.676	93.927	93.977	99.576	94.211	93.587
	*A. thaliana*	99.925	97.164	92.815	92.443	99.763	93.153	93.747
	*C. elegans*	99.994	94.841	95.342	93.925	99.779	95.168	93.665
	*H. sapiens*	99.999	96.381	92.393	92.896	–	94.143	91.738

^a^GraphMap always appears core dumped information for *H. sapiens* dataset, it does not output the results

**Table 3:** ACR standard deviation (std) of seven methods on four real datasets

	Datasets	smsMap	lordFAST	BLASR	BWA-MEM	GraphMap^a^	minimap2	NGLMR
std	*E. coli*	5.73E-5	0.09208	0.15266	0.15370	0.04106	0.14725	0.14089
	*A. thaliana*	0.01653	0.13360	0.19902	0.20301	0.01688	0.19362	0.15433
	*C. elegans*	0.00466	0.18524	0.14132	0.15507	0.01775	0.14245	0.14936
	*H. sapiens*	0.00001	0.14932	0.17926	0.16256	–	0.14897	0.17192

^a^GraphMap always appears core dumped information for *H. sapiens* dataset, it does not output the results

**Fig. 2:**
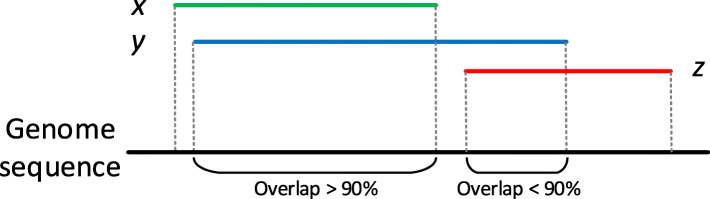
A toy example to illustrate the covering and non-covering alignments. *x*, *y* and *z* are different alignments obtained by different methods for the same read. We can see that alignments *x* and *y* cover each other as aligned regions on the reference genome share more than 90% overlap. On the other hand, the alignment *z* does not cover either alignment *x* or *y*

Additionally, the agreement between different methods based on their alignment results were measured. For a given read, there are two alignment results *x* and *y* generated by two methods. We define *x* covers *y* if the aligned region on the reference genome covered by *x* shares at least 90% overlaps with the aligned region covered by *y* [27]. Figure 2 presents the illustration of covering and non-covering alignments. Table 4 reports how best alignments from different methods cover each other for *E. coli* dataset. Specifically, each row contains the percentage of alignments generated by one method that covers alignments obtained by other tools. For example, among all aligned reads for smsMap and BLASR in Table 4, 95.23% of the alignments produced by BLASR are covered by smsMap, while only 85.76% of the alignments generated by smsMap are covered by BLASR. Tables S12, 13, 14 report the agreement between different methods on *A. thaliana*, *C. elegans,* and *H. sapiens* datasets. We can see that the alignment results of smsMap give a high coverage of the alignments obtained by other methods. With a lack of the true mappings for these four real datasets, the consensus results in Tables 4 and S12, 13, 14 show some extra support for the fact that the alignments of smsMap are reliable.

**Table 4:** Agreement of different alignment methods for *E. coli* dataset

	smsMap	lordFAST	BLASR	BWA-MEM	GraphMap	minimap2	NGMLR
smsMap	N/A	75.44	85.76	85.46	96.75	84.26	82.31
lordFAST	87.33	N/A	78.61	83.20	97.27	83.70	80.53
BLASR	95.23	74.70	N/A	93.56	97.90	92.14	90.76
BWA-MEM	93.26	76.81	89.96	N/A	98.58	94.50	91.37
GraphMap	85.35	74.09	78.14	83.69	N/A	82.02	80.78
minimap2	93.86	78.04	91.79	97.01	98.71	N/A	92.99
NGLMR	92.52	75.13	91.82	95.19	97.63	94.25	N/A

Each row shows the percentage of best alignment from the corresponding method that covers alignments from other methods. Note that this table is dissymmetric

Given the massive sequences generated by SMS technology, we also need to consider the computational complexity of the mapping tools. To demonstrate the computational efficiency of our smsMap, we compared smsMap with other mapping tools on the four real-world datasets. Table 5 shows the running time (wall-time) and memory usage by using the seven tools. We can see that for the reference genome datasets with small size and relatively short average read lengths, such as *E. coli* and *A. thaliana* datasets, the speed of smsMap is a little lower than minimap2 and lordFAST. But for the *H. sapiens* genome dataset with larger size and relatively short average read length, smsMap is faster than lordFAST, BWA-MEM, NGMLR, and BLASR. These results indicate that smsMap is efficient to align SMS reads, especially for the larger size of the reference genome.

**Table 5:** Running time (min) and memory usage (GB) of each mapping method on three datasets

		smsMap	BWA-MEM	BLASR	lordFAST	minimap2	GraphMap*	NGMLR
*E. coli*	Time	0.83	0.85	1.28	0.27	0.17	0.95	1.32
	Memory	2.34	1.29	0.51	1.84	2.44	0.88	11.234
*A. thaliana*	Time	3	32	24	3	2	6	12
	Memory	2.56	1.25	3.58	2.03	4.94	3.19	11.45
*C. elegans*	Time	10	87	61	10	2	24	25
	Memory	2.52	2.11	3.58	2.04	8.53	3.18	11.60
*H. sapiens*	Time	9	69	63	30	4	–	17
	Memory	9.34	6.48	26.71	6.84	11.16	–	15.78

*Due to that GraphMap always appears core dumped information for *H. sapiens* dataset, it does not output the results

## Discussion

Most mapping methods for SMS reads adopt the classical seed-and-extension methodology to obtain the alignment results. That is, they first find the exactly matched seeds in the reference genome, then select the candidate aligned region based on counting the number of matched seeds (e.g., rHAT and lordFAST) or chaining a group of seeds that are co-linear or close to each other (e.g., BLASR, LAMSA, GraphMap, NGMLR, BWA-MEM, and minimap2), finally, extend the alignment to the non-seed fragments within the selected candidate regions. However, the candidate aligned region of each query read is always a part of the read and cannot completely cover the whole read length. As a result, these methods usually focus on producing local mapping results for the query read, other than obtaining the whole end-to-end alignment, leading to low aligned coverage.

To solve the above issue, here we developed smsMap to obtain the whole read alignment by locating the alignment starting positions. smsMap is also a seeds-based method using BWT-FM index technique, there are two differences between smsMap and other methods: i) smsMap proposes a scoring strategy to select the candidate aligned regions by defining a credibility function to measure the starting position credibility, which can locate the aligned positions for each query read; ii) smsMap introduces a banded alignment on the low column memory matrix to get the alignment results of the whole read. The credibility function ensures that smsMap can locate the aligned positions for every query read, even in the situation that the matched seeds are dispersedly distributed in the reference genome. Thus, smsMap can get higher FAR, that is, align more reads. The banded alignment with the low column can obtain the whole end-to-end alignment, not local alignment achieved by other methods. Therefore, the FAB of smsMap is higher than other methods. Table S15 shows the example alignments of different methods for one sequence with length of 296 bp, the detail base-to-base alignments are also provided in the supplementary file. We can see that smsMap aligned the whole read, while other tools failed to align the whole read. So, the alignment of smsMap can truly reflect the error rate of the sequencing platform, while other tools just output the local alignment results. Now, smsMap just outputs the best-aligned position on the reference for query reads. But for a long chimeric read that a part of the read comes from one position and another part of the read from a different position. smsMap still reports one aligned position. This is a limitation of smsMap. Thus, if users prefer to get the whole end-to-end alignments, smsMap is recommended, if users prefer to obtain other aligned positions for a query read, other methods such as lordFAST and minimap2 are recommended.

## Conclusions

With the development of SMS technologies (e.g., PacBio and Oxford Nanopore MinION) that produce long but noisy reads, mapping these reads to the reference genome has become a central bioinformatics challenge. It is important to develop novel long read alignment tools with better aligning accuracy as well as higher aligned coverage.

In this article, we developed smsMap to improve mapping quality of the long reads. Mainly, there are two key features of smsMap. i) smsMap utilizes a strategy to identify the starting positions in the query read and reference genome by designing a position credibility function, this strategy makes more query reads aligning on the genome, and also enables more segments of the query read mapping to the genome. ii) Compared with the traditional banded alignment algorithm, smsMap implements the banded aligning on a low column matrix, which can reduce the memory usage. The experimental results on both simulated and real-life SMS datasets show that smsMap achieves higher aligned read coverage ratio and better mapping quality, and it can be more robust to the high sequencing errors. In addition, smsMap adopts the strategy of locating the start position that provides the split alignments of the reads. It makes smsMap appropriate for aligning reads deriving from regions with long structural variations.

## Methods

**Fig. 3:**
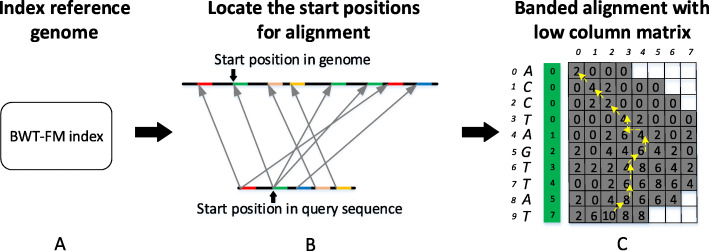
Overview of the smsMap mapper. **a** Index the reference genome by BWT-FM strategy. **b** Locate the starting positions in the genome and the query read, exact word matches are shown with different colors. **c** A detailed dynamic alignment result is obtained by using the proposed column reduction banded alignment as a guide. The backtracking route is denoted by the yellow arrows

An overview of the smsMap mapper is shown in Fig. 3. smsMap mapper mainly includes three main phases: i) build the BWT-FM index of the reference genome (Fig. 3a), ii) locate the best starting positions in genome and query read by designing a position location approach (Fig. 3b), and iii) obtain the detailed dynamic alignment results by presenting a strategy of banded alignment on the low column memory matrix (Fig. 3c).

### Indexing the reference genome

The BWT-FM index is a compressed full-text substring index based on the BWT transformation, which can efficiently find the number of occurrences of a substring within the compressed text, as well as locate the position of each occurrence [39]. It has been widely applied in bioinformatics, such as whole-genome alignment [40], short DNA sequence mapping [13], etc. Inspired by the BWT-FM index that allows long reference genome to be searched efficiently with low memory usage [41], here, we use the BWT-FM technique implemented in *combined-index* [27] to construct the index for the reference genome, which can quickly locate and find the match positions in the reference genome for a given short word (default word length *k* = 14).

### Locating the starting positions for alignment

Suppose that the query read *r* is the input sequence, where we do not know which position in the reference genome that *r* comes from or which region in the genome that has high similarity to *r*. It is impossible to align *r* with each subsequence of the genome due to high time complexity. Therefore, it is crucial to design a searching strategy to quickly locate the positions in query read *r* and genome for mapping.

**Fig. 4:**
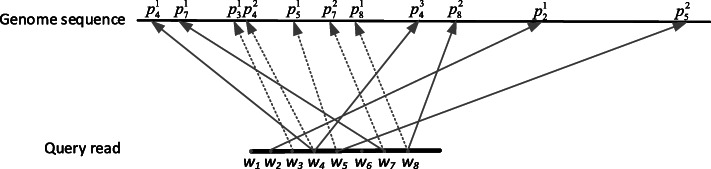
The mapping positions for a query read of SMS. Some words (e.g., *w*_*1*_ and *w*_*6*_) cannot be mapped to the genome because it contains sequencing errors, the right mapping positions for alignment are dotted

Because the genome sequence is greatly longer than the query read, there is a high possibility that one short word in the query read can be found in multi-positions in the genome, but only one of the multi-positions for each word is the right location. In addition, due to the query reads generated by SMS technology containing different errors, there is a high possibility that some words cannot be mapped to the genome (e.g., word *w*_1_ and *w*_6_ in Fig. 4), and the widths between the corresponding mapped positions of adjacent word pairs are usually unequal (e.g., ${p}_4^2-{p}_3^1\kern0.5em \ne \kern0.5em {p}_8^1-{p}_7^2$ in Fig. 4). Considering these issues, we proposed the following position location strategy to find the starting positions for aligning quickly.

First, we search all exact short word matches from the query read *r* in the genome index built in the previous step. An exact match of word *w*_*i*_ in the read *r* onto the genome can be described by a position set *W*_*i*_:

3 $$ {W}_i=\left({p}_i^1,{p}_i^2,\dots {p}_i^l,\dots {p}_i^{L_i},{o}_i^1,{o}_i^2,\dots {o}_i^l,\dots {o}_i^{L_i}\right) $$

where *i* is the position of the *i*-th word (*w*_*i*_) in the read *r*, ${p}_i^l\left(l=1,2,\cdots, {L}_i\right)$ is the *l*-th matched position of *w*_*i*_ on the genome, ${o}_i^l=\left({p}_i^l-i\right)$ is the modified position of the *l*-th matched position.

After building the position sets for all the matched words in read *r*, we can define the following credibility function $S\left({o}_i^l\right)$ to measure the starting position credibility for aligning.

4 $$ S\left({o}_i^l\right)=\sum \limits_{j=1}^{\Theta}\sum \limits_{k=1}^{L_j}\delta \left(\left|{o}_j^k-{o}_i^l\right|\right) $$

5 $$ \delta \left(\left|{o}_j^k-{o}_i^l\right|\right)=\left\{\begin{array}{l}1,\kern0.5em if\left|{o}_j^k-{o}_i^l\right|\le L(r)\kern0.5em \\ {}0,\kern0.5em otherwise\end{array}\right. $$

where Θ is the total matched word number in the query read *r*, *L*_*j*_ is the total matched word number of word *w*_*j*_ on the genome, and *L*(*r*) is an error-tolerant length function for the query read *r* (here we set *L*(*r*) = 0.2*r*).

From all the credibility scores of ${o}_i^l\left(i=1,2,\cdots, \Theta, l=1,2,\cdots, {L}_i\right)$, we select the ${o}_i^l$ with largest score value to identify the alignment starting positions on the read and genome, that is, the position of word *w*_*i*_ on the read is considered as the alignment starting position, and its *l*-th matched position on the genome is considered as the alignment starting position.

### Banded alignment on the low column memory matrix

**Fig. 5:**
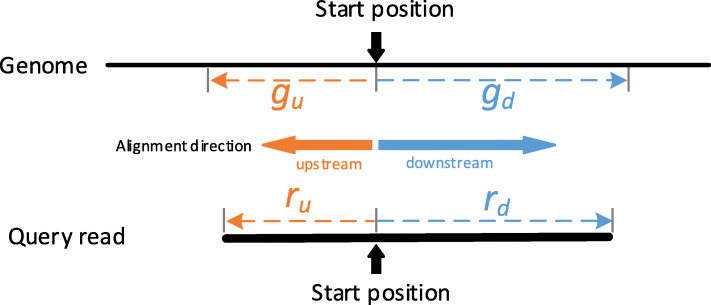
Starting alignment positions on the read and genome and the four split segments of *g*_*d*_, *g*_*u*_, *r*_*d*_ and *r*_*u*_. Generally, the length of *g*_*u*_ and *g*_*d*_ are 1.2 times of *r*_*u*_ and *r*_*d*_ length

**Fig. 6:**
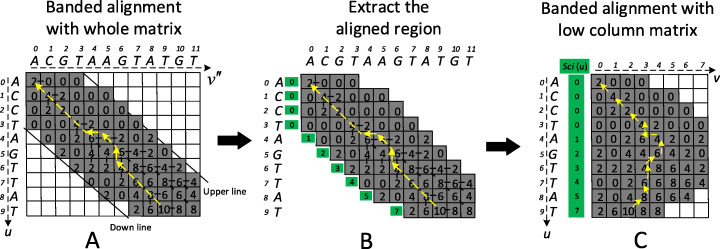
Banded alignment flowchart from the traditional full matrix to the low column matrix. **a** Traditional banded alignment on the full matrix. The upper line and bottom line are the alignment boundary. **b** Extract the alignment region. Numbers in green boxes are the started column index of the full memory matrix. **c** Banded alignment on the low column matrix in which the yellow line is the backtracking path

After identifying the alignment starting positions on the read and genome, the starting positions generally divide the query read into downstream segment *r*_*d*_ and upstream segment *r*_*u*_, the genome into downstream segment *g*_*d*_ and upstream segment *g*_*u*_ (Fig. 5). For aligning each pair of segments (i.e., pair of *r*_*d*_ and *g*_*d*_, or *r*_*u*_ and *g*_*u*_), the traditional banded alignment dynamic programming [42] can be applied to get the alignment result (Fig. 6a). Evidently, it needs a *l*(*r*_*d*_) × *l*(*g*_*d*_) matrix to store the alignment scores, where *l*(*r*_*d*_) and *l*(*g*_*d*_) are the length of *r*_*d*_ and *g*_*d*_, respectively. Generally, the length of *l*(*r*_*d*_) and *l*(*g*_*d*_) are over 1000 bps, and it requires bigger memory usage. In order to reduce the matrix memory usage, here we present the following strategy to reduce the matrix column size for relieving the large memory usage (Fig. 6b and c).

We first extract the score region and index for every column in Fig. 6a. The number (i.e., starting column index) in green boxes in Fig. 6b can be used to obtain the bases in the genome for scoring. Based on the observation from Fig. 6b that the maximum aligning base number for each row is 2*b* (*b* is the bandwidth), we can use a matrix with *l*(*r*_*d*_) × 2*b* (Fig. 6c) to store the aligning scores in Fig. 6b. Because 2*b* is much smaller than *l*(*g*_*d*_), the matrix *l*(*r*_*d*_) × 2*b* can significantly reduce memory usage. The scores in the low column matrix in Fig. 6c are calculated by the following eqs. 6–10.

6 $$ {\displaystyle \begin{array}{c}F\left(u,v\right)=\max \left\{\begin{array}{l}F\left(u-1,v\hbox{'}-1\right)+ Score\left[{r}_d(u),{g}_d\left(v\hbox{'}\hbox{'}\right)\right]\\ {}F\left(u-1,v\hbox{'}\right)-d\\ {}F\left(u,v\hbox{'}-1\right)-d\end{array}\right.\\ {}u\in \left[0,\kern1em l\left({r}_d\right)\right]\kern0.5em ,v\in \left[0,\kern0.5em 2b\right]\end{array}} $$

7 $$ v\hbox{'}=v+ sci(u)- sci\left(u-1\right) $$

8 $$ v\hbox{'}\hbox{'}=v+ sci(v) $$

9 $$ sci(u)=\max \left[ floor\left({l}_{down}(u)\right),\kern0.5em 0\right] $$

10 $$ {l}_{down}(u)=1.2\times u-b $$

where *F*(*u*, *v*) is the score value of the *u*-th row and *v*-th column in the matrix in Fig. 6c, *Score*[*r*_*d*_(*u*), *g*_*d*_(*v* ' ')] is the match score or mismatch penalty for the *u*-th base in segment *r*_*d*_ and the *v* ' '-th base in *g*_*d*_, *sci*(*u*) is the starting column index and *floor*() is the floor function. Here we define the bandwidth *b* = *αl*_*r*_, where *l*_*r*_ is the read length, *α* is a width coefficient. The default value of *α* is 0.1, which can cover almost all aligned paths (see supplementary file for more discussion about the banded width).

After obtaining the two low column matrices of *l*(*r*_*d*_) × 2*b* and *l*(*r*_*u*_) × 2*b*, we applied the banded alignment algorithm on these two matrices to align *r*_*d*_ with *g*_*d*_, and *r*_*u*_ with *g*_*u*_, respectively. In the end, we combine the aligning results of downstream segments (i.e., *r*_*d*_ with *g*_*d*_) and upstream segments (i.e., *r*_*u*_ with *g*_*u*_) to get the mapping result of the query read and the reference genome.

## Supplementary information

**Additional file 1: Figure S1.** An example to explain how to calculate the metrics of cFAR, cFAB and cARC. The numbers above each read are the start and end aligned positions. **Figure S2.** The base-to-base alignments for different methods with one sequence (length: 296 bp). lordFAST and minimap2 fail to align this sequence. The statistics of the alignments are listed in Table S15. **Figure S2.** Diagram of using three bandwidths for aligning. (A) Too large bandwidth. (B) Too small bandwidth. (C) Appropriate bandwidth, it not only covers the backtracking, but also reduces the memory usage. **Figure S3.** The distribution of width coefficient for simulated datasets with different error rates ranging from 5 to 30%. **Table S1**. Running command lines of different mapping programs. **Table S2.** Parameter settings of NPBSS for generating simulated datasets. **Table S3.** Read number and total bases of simulated datasets. **Table S4.** The cFAR, cFAB and cACR for different methods on the simulated datasets. **Table S5.** Alignment scores of different methods for *E. coli* simulated datasets. The values in the brackets are the min. and max. Alignment scores. **Table S6.** The cFAR, cFAB and cACR for different methods on the simulated datasets of *H. sapiens* (CHM1). **Table S7.** Different SVs types and its breakpoints in the genome. **Table S8.** PacBio datasets website links. **Table S9.** Reference genome website links. **Table S10.** Statistics of *E. coli*, *A. thaliana*, *C. elegans* and *H. sapiens* datasets. **Table S11.** FAR(%), FAB(%) and ACR(%) of eight methods on *E. coli* UTI89 dataset. **Table S12.** Agreement of different alignment methods for *A. thaliana* dataset. **Table S13.** Agreement of different alignment methods for *C. elegans* dataset. **Table S14.** Agreement of different alignment methods for *H. sapiens* dataset. **Table S15.** Aligned results of different alignment methods for one sequence. (see Figure S2 for detail alignments). **Table S16.** The average and maximum of width coefficient for simulated datasets.

## Data Availability

All data in this paper is available in the supplementary file or from the corresponding author on a reasonable request.

## Abbreviations

SMS: Single Molecule Sequencing
HTS: High-throughput sequencing
SVs: Structural variations
BWT-FM: Burrows-Wheeler Transform Full-text Minute-space
SDP: Sparse dynamic programming
RHT: Regional hash table
DAG: Direct acyclic graph
cFAR: The fraction of correctly aligned reads
cFAB: The fraction of correctly aligned bases
cACR: The average coverage ratio of correctly aligned reads
FAR: The fraction of aligned reads
FAB: The fraction of aligned bases
ACR: Average coverage ratio of aligned reads
