# Supplementary material for: smsMap: mapping single molecule sequencing reads by locating the alignment starting positions
Source: BMC Bioinformatics. 2020 Aug 4;21:341. doi: 10.1186/s12859-020-03698-w (PMC7430848; doi:10.1186/s12859-020-03698-w)
Supplement: Supplementary file 1 — Additional file 1: Figure S1. An example to explain how to calculate the metrics of cFAR, cFAB and cARC. The numbers above each read are the start and end aligned positions. Figure S2. The base-to-base alignments for different methods with one sequence (length: 296 bp). lordFAST and minimap2 fail to align this sequence. The statistics of the alignments are listed in Table S15. Figure S2. Diagram of using three bandwidths for aligning. (A) Too large bandwidth. (B) Too small bandwidth. (C) Appropriate bandwidth, it not only covers the backtracking, but also reduces the memory usage. Figure S3. The distribution of width coefficient for simulated datasets with different error rates ranging from 5 to 30%. Table S1. Running command lines of different mapping programs. Table S2. Parameter settings of NPBSS for generating simulated datasets. Table S3. Read number and total bases of simulated datasets. Table S4. The cFAR, cFAB and cACR for different methods on the simulated datasets. Table S5. Alignment scores of different methods for E. coli simulated datasets. The values in the brackets are the min. and max. Alignment scores. Table S6. The cFAR, cFAB and cACR for different methods on the simulated datasets of H. sapiens (CHM1). Table S7. Different SVs types and its breakpoints in the genome. Table S8. PacBio datasets website links. Table S9. Reference genome website links. Table S10. Statistics of E. coli, A. thaliana, C. elegans and H. sapiens datasets. Table S11. FAR(%), FAB(%) and ACR(%) of eight methods on E. coli UTI89 dataset. Table S12. Agreement of different alignment methods for A. thaliana dataset. Table S13. Agreement of different alignment methods for C. elegans dataset. Table S14. Agreement of different alignment methods for H. sapiens dataset. Table S15. Aligned results of different alignment methods for one sequence. (see Figure S2 for detail alignments). Table S16. The average and maximum of width coefficient for simulated datasets. [file 12859_2020_3698_MOESM1_ESM.docx]

**smsMap: mapping single molecule sequencing reads by locating the alignment starting positions**

Ze-Gang Wei^1,2^, Shao-Wu Zhang^1*^, Fei Liu^2^

1 Key Laboratory of Information Fusion Technology of Ministry of Education, School of Automation, Northwestern Polytechnical University, Xi’an 710072, China

2 Institute of Physics and Optoelectronics Technology, Baoji University of Arts and Science, Baoji 721016, China

* Corresponding author. Emails: zhangsw@nwpu.edu.cn

**Supplementary file**

**Calculating cFAR, cFAB and cACR**

Following Figure S1 shows an example to explain how to calculate the cFAR, cFAB and cACR. Here we have two reads (*r_1_* and *r_2_*) for mapping:

*r_1_*=*TCCTGTGATCTCTTAGGGAACCGTAGCTGGCTTCAACTACATG*

*r_2_*=*CTCATAACTTCACTGGCTATCGAGCCTCGAATGTCTACTTGGACT*

The lengths of *r_1_* and *r_2_* are 43 bp and 45 bp, respectively. The alignment results of *r_1_* and *r_2_* generated by one method are shown in Figure 1. Suppose that *r_1_* and *r_2_* were aligned to the true strand. For *r_1_*, the aligned bases number is 40 (start and end aligned positions are 3 and 42). For *r_2_*, the aligned bases number is 29 (start and end aligned positions are 15 and 43). Based on the definition of cFAR, the *r_2_* is not a correctly aligned read since the aligned bases are less than 0.9**len*(*r_2_*), so the cFAR = 1/2=0.5. For the cFAB, the number of matched bases of *r_1_* is 34, and suppose that all the matched bases are within *T* bp (here *T*=5) of the corresponding truth positions on the genome, thus, the number of correct matched bases is 34. As a result, the cFAB=34/34=100%. Finally, the cACR is the aligned region divided by the whole read length, that is cACR=40/45=88.89%. We have provided this explanation in the supplementary file to help readers understand how to calculate the cFAR, cFAB and cACR.

**Figure S1.** An example to explain how to calculate the metrics of cFAR, cFAB and cARC. The numbers above each read are the start and end aligned positions.

**An example of base-to-base alignments for different methods (the sequence length is 296 bp)**


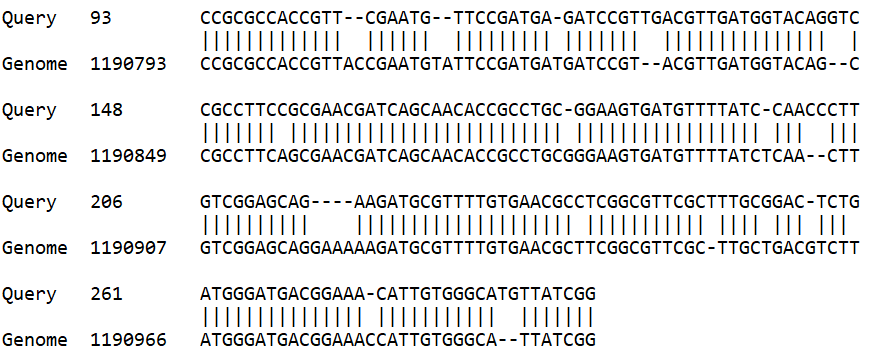

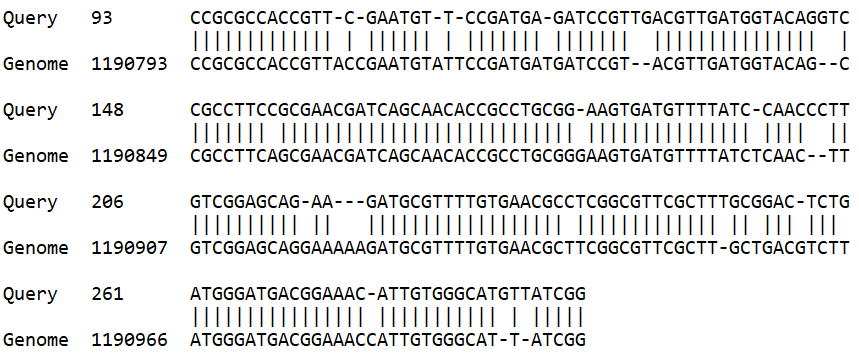


(a) BWA-MEM (b) GraphMap


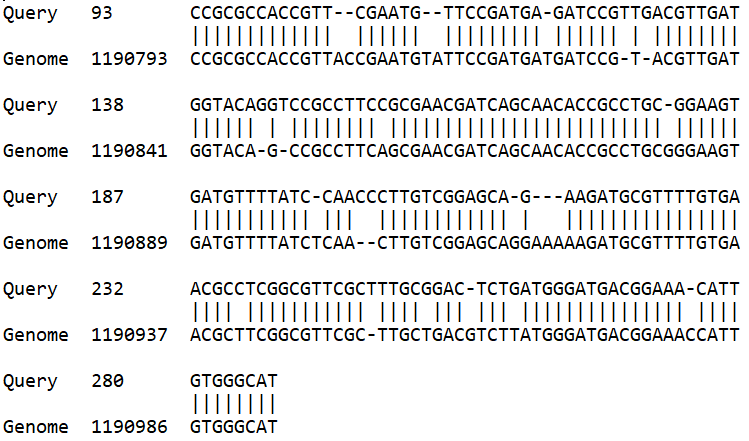

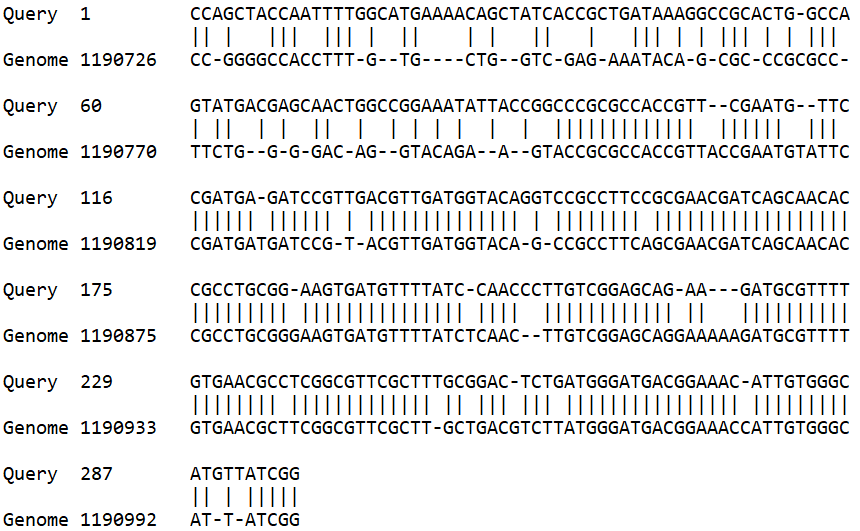


(c) BLASR (d)smsMap


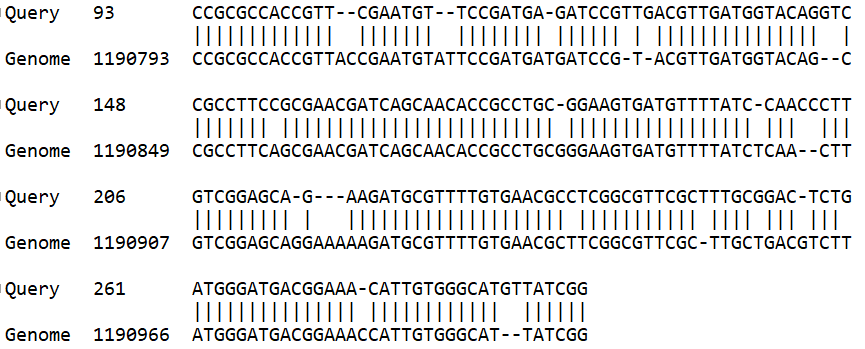


(e) NGMLR

**Figure S2.** The base-to-base alignments for different methods with one sequence (length: 296 bp). lordFAST and minimap2 fail to align this sequence. The statistics of the alignments are listed in Table S15.

**Banded width**

In the alignment phase, smsMap uses a banded alignment approach with the low column matrix to relieve the large memory usage for aligning the longer segments. The bandwidth (*b*) will affect the alignment results. As illustrated in Figure S2-A, if *b* is too large, the size of the score matrix will be almost the same as the original matrix, resulting in more memory usage. If *b* is too small (Figure S2-B), it will not cover the best aligned backtracking path (red lines with arrows), resulting in inaccuracy alignment result. In this paper, we defined the bandwidth $b=\alpha l_{r}$, where $l_{r}$ is the read length, $\alpha$is a width coefficient. Figure S3 shows the histogram distribution of width coefficient for *E. coli* simulated datasets with error rates ranging from 5% to 30% after aligning them to the true position on the genome. We can observe that the banded width presents similar distribution (Gaussian normal curve) with different error rates. Table S16 lists the average and maximum of width coefficient. We can see that the smsMap can cover almost aligned path when the width coefficient was set as 0.1.

**Figure S2**. Diagram of using three bandwidths for aligning. (A) Too large bandwidth. (B) Too small bandwidth. (C) Appropriate bandwidth, it not only covers the backtracking, but also reduces the memory usage.


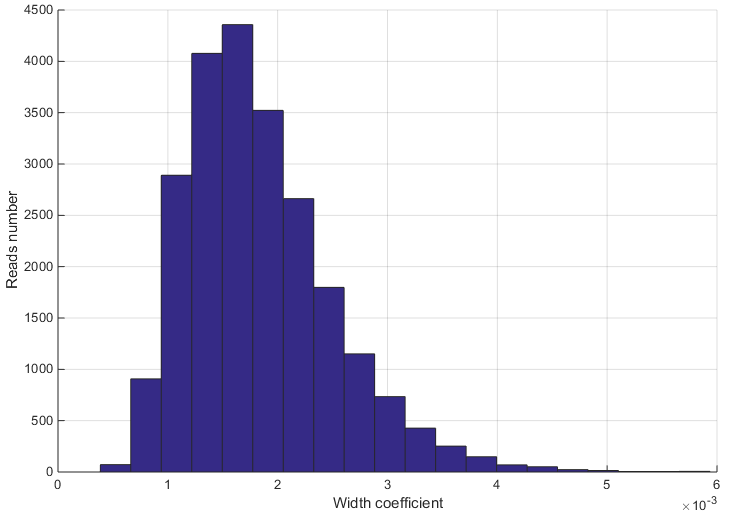

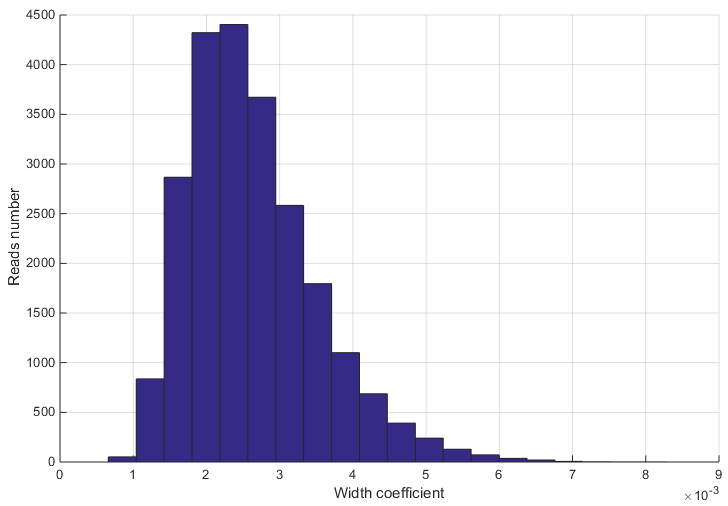


(a) 5% error rate (INDELerror rate: 4%) (b) 10% error rate (INDELerror rate: 8%)


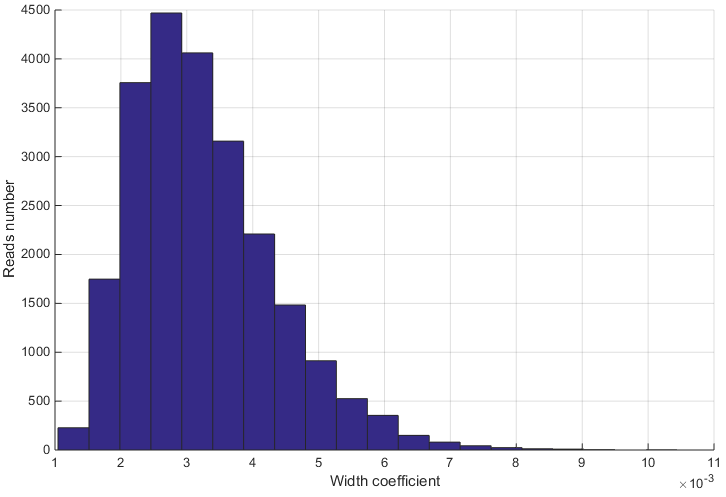

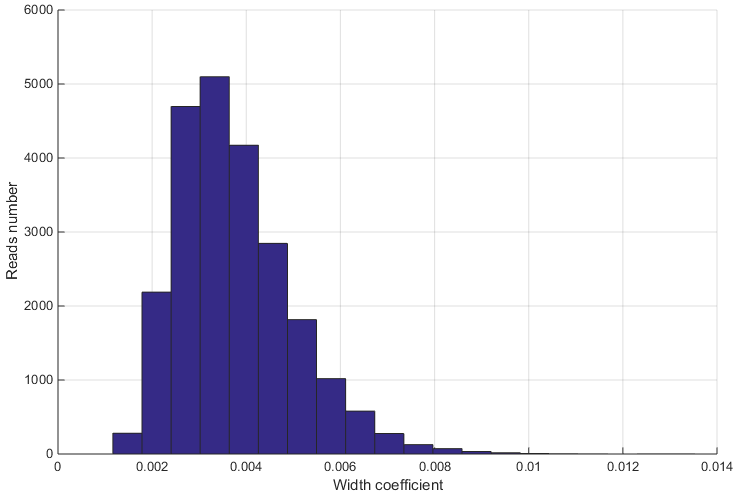


(c) 15% error rate (INDELerror rate: 12%) (d) 20% error rate (INDELerror rate: 14%)


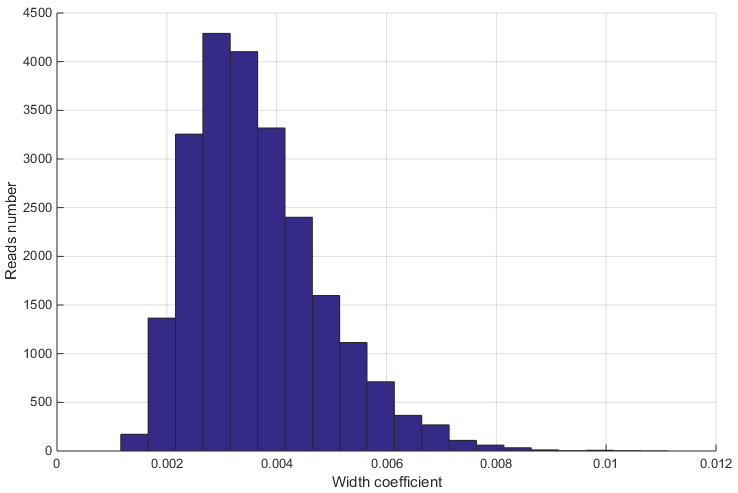

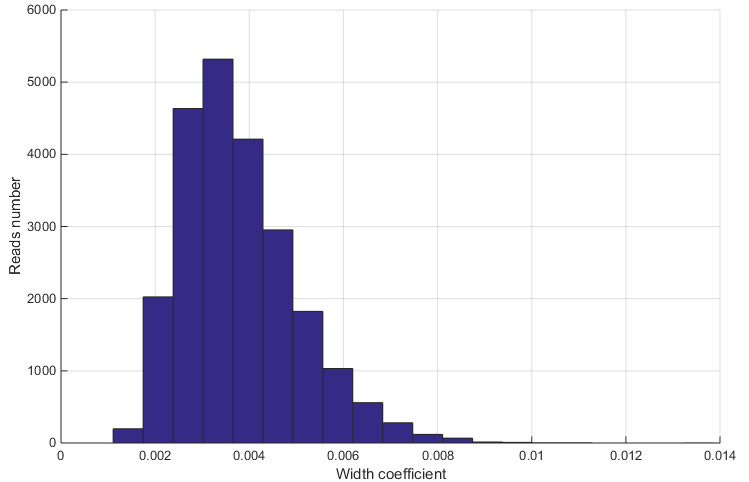


(e) 25% error rate (INDELerror rate: 20%) (f) 30% error rate (INDELerror rate: 24%)

**Figure S3**. The distribution of width coefficient for simulated datasets with different error rates ranging from 5% to 30%.

**Tables**

**Table S1**. Running command lines of different mapping programs.

| Programs | Running command lines |
| --- | --- |
| smsMap | locate --index genome.fa; locate -t 16 --search genome.fa --seq seq.fa >pos.txt |
|  | smsMap --seq seq.fa --genome genome.fa --pos pos.txt --out aligned.txt |
| BLASR | blasr sequences.fa genome.fa -m 0 --bestn 1 --nproc 16 >blasr_aligned.txt |
| minimap2 | minimap2 -t 16 -a genome.fa sequence.fa > minimap2.sam |
| GraphMap | graphmap align -r genome.fa -d sequence.fa -o graphmap_aligned.sam |
| NGMLR | ngmlr -t 16 -r genome.fa -q sequence.fa -o ngml.sam |
| lordFAST | lordfast --index genome.fa |
|  | lordfast -t 16 --search genome.fa --seq sequence.fa > map.sam |
| rHAT | rHAT-indexer ./ ref.fasta |
|  | rHAT-aligner ./ reads.fa ref.fa > rhat.sam |
| BWA-MEM | bwa index genome.fa |
|  | bwa mem -t 16 -x pacbio genome.fasta sequence.fasta > bwa_mem.sam |

**Table S2**. Parameter settings of NPBSS for generating simulated datasets.

| Error rates (%) | Command lines |
| --- | --- |
| 5 | NPBSS('genome.fa', '-dep 50 -len 10000 -sub 0.01 -ins 0.02 -del 0.02') |
| 10 | NPBSS('genome.fa', '-dep 50 -len 10000 -sub 0.02 -ins 0.04 -del 0.04') |
| 15 | NPBSS('genome.fa', '-dep 50 -len 10000 -sub 0.03 -ins 0.06 -del 0.06') |
| 20 | NPBSS('genome.fa', '-dep 50 -len 10000 -sub 0.06 -ins 0.07 -del 0.07') |
| 25 | NPBSS('genome.fa', '-dep 50 -len 10000 -sub 0.05 -ins 0.10 -del 0.10') |
| 30 | NPBSS('genome.fa', '-dep 50 -len 10000 -sub 0.06 -ins 0.12 -del 0.12') |

**Table S3**. Read number and total bases of simulated datasets.

| Error rates (%) | Sequencing depth | Number of reads | Total bases |
| --- | --- | --- | --- |
| 5 | 50 | 23,131 | 227,711,513 |
| 10 |  | 23,131 | 226,892,435 |
| 15 |  | 23,123 | 225,519,916 |
| 20 |  | 23,136 | 227,351,278 |
| 25 |  | 23,125 | 227,086,282 |
| 30 |  | 23,119 | 227,500,110 |

**Table S4**. The cFAR, cFAB and cACR for different methods on the simulated datasets.

|  | Error rate (%) | smsMap | lordFAST | BLASR | BWA-MEM | GraphMap | minimap2 | NGLMR | rHAT |
| --- | --- | --- | --- | --- | --- | --- | --- | --- | --- |
|  | 5 | 100 | 99.9741 | 100 | 100 | 100 | 100 | 99.9741 | 99.9914 |
|  | 10 | 100 | 99.9654 | 100 | 100 | 99.9870 | 100 | 99.9568 | 99.9870 |
| cFAR | 15 | 100 | 99.9611 | 99.9784 | 99.9914 | 99.9914 | 99.9697 | 99.9568 | 99.9957 |
|  | 20 | 99.9987 | 99.9568 | 99.1528 | 99.9092 | 99.9957 | 99.6024 | 99.9568 | 99.9870 |
|  | 25 | 99.9961 | 99.9481 | 91.2822 | 99.2865 | 99.9784 | 97.0897 | 99.7103 | 99.9914 |
|  | 30 | 99.9952 | 99.5718 | 59.2370 | 95.6356 | 99.9870 | 87.1318 | 97.6772 | 99.8702 |
|  | 5 | 100 | 99.9693 | 99.9778 | 99.9461 | 99.9988 | 99.9875 | 99.8092 | 99.7058 |
|  | 10 | 100 | 99.8959 | 99.9570 | 99.9331 | 99.9956 | 99.9766 | 99.7370 | 99.6006 |
| cFAB | 15 | 100 | 99.8936 | 99.8959 | 99.9236 | 99.9815 | 99.9526 | 99.5958 | 99.5495 |
|  | 20 | 99.9976 | 99.8795 | 99.0187 | 99.8050 | 99.9856 | 99.7209 | 99.2075 | 99.3822 |
|  | 25 | 99.9923 | 99.8543 | 90.5825 | 99.2907 | 99.9767 | 98.2092 | 98.4858 | 98.6970 |
|  | 30 | 99.9718 | 99.5926 | 56.2189 | 96.2055 | 99.9314 | 89.9870 | 94.3840 | 94.7698 |
|  | 5 | 100 | 99.9786 | 99.9686 | 99.9533 | 99.9983 | 99.9817 | 99.7705 | 99.8043 |
|  | 10 | 100 | 99.9466 | 99.9380 | 99.9386 | 99.9971 | 99.9655 | 99.7288 | 99.7556 |
| cACR | 15 | 100 | 99.9199 | 99.8694 | 99.9245 | 99.9957 | 99.8566 | 99.5894 | 99.6871 |
|  | 20 | 99.9982 | 99.9044 | 99.5734 | 99.8340 | 99.9689 | 99.5876 | 99.1708 | 99.5410 |
|  | 25 | 99.9824 | 99.8957 | 98.5078 | 99.5535 | 99.9535 | 98.2453 | 98.2481 | 98.9281 |
|  | 30 | 99.9657 | 99.8754 | 94.3428 | 98.5162 | 99.9222 | 99.1441 | 94.6032 | 95.5194 |
|  | 5 | 100 | 99.9220 | 99.9464 | 99.8994 | 99.997 | 99.9692 | 99.5543 | 99.5021 |
|  | 10 | 100 | 99.8080 | 99.8950 | 99.8717 | 99.979 | 99.9421 | 99.4235 | 99.3442 |
| Sensitivity | 15 | 100 | 99.7747 | 99.7438 | 99.8395 | 99.9686 | 99.7790 | 99.1440 | 99.2337 |
|  | 20 | 99.9945 | 99.7409 | 97.7609 | 99.5488 | 99.9502 | 98.9148 | 98.3423 | 98.9131 |
|  | 25 | 99.9708 | 99.6983 | 81.4518 | 98.1420 | 99.9086 | 93.6778 | 96.4801 | 97.6306 |
|  | 30 | 99.9327 | 99.0425 | 31.4184 | 90.6415 | 99.8406 | 77.7362 | 87.2162 | 90.4060 |
|  | 5 | 100 | 99.9479 | 99.9464 | 99.8994 | 99.9971 | 99.9692 | 99.5801 | 99.5106 |
|  | 10 | 100 | 99.8425 | 99.8950 | 99.8717 | 99.9927 | 99.9421 | 99.4665 | 99.3571 |
| Precision | 15 | 100 | 99.8135 | 99.7654 | 99.8481 | 99.9772 | 99.8092 | 99.1868 | 99.2380 |
|  | 20 | 99.9958 | 99.7840 | 98.5962 | 99.6393 | 99.9545 | 99.3096 | 98.3848 | 98.9260 |
|  | 25 | 99.9747 | 99.7501 | 89.2308 | 98.8473 | 99.9302 | 96.4859 | 96.7604 | 97.6390 |
|  | 30 | 99.9375 | 99.4685 | 53.0384 | 94.7780 | 99.8536 | 89.2168 | 89.2902 | 90.5235 |

**Table S5.** Alignment scores of different methods for *E. coli* simulated datasets. The values in the brackets are the min. and max. alignment scores.

| Error rate (%) | 5 | 10 | 15 | 20 | 25 | 30 |
| --- | --- | --- | --- | --- | --- | --- |
| smsMap | 17747 (1308,87198) | 16725 (1227,81422) | 15522 (910, 81151) | 14431 (464, 72291) | 13489 (323, 68822) | 12555  (321, 68150) |
| lordFAST | 18306 (330,87834) | 17468 (378, 83968) | 16565 (134,82660) | 16230 (164,80042) | 15275   (252,74946) | 14745 (200, 72734) |
| BLASR | 17782  (1293,87192) | 16749 (1159,81422) | 15532 (894,81143) | 14425 (103,72261) | 13479 (86,68784) | 12306 (89,67981) |
| BWA-MEM | 18389 (1386,87846) | 17676 (1290,84418) | 16950 (1106,82870) | 16802 (792,81606) | 16135 (1192,77028) | 15940 (1288,75548) |
| GraphMap | 18299 (1374,87834) | 17474 (1290,83950) | 16568 (1060,82654) | 16238 (768,80036) | 15291 (1122,74904) | 14764 (608,72668) |
| Minimap2 | 18387 (1380,87846) | 17655 (1290,84385) | 16890 (1384,82864) | 16707 (1236,81188) | 16098 (1512,76878) | 16038 (1404,74912) |
| NGLMR | 18273 (1336,87834) | 17440 (1258,83956) | 16513 (1046,82656) | 16111  (750, 80012) | 15094 (1106,74844) | 14265  (872, 72692) |
| rHAT | 18101  (58, 83776) | 17101 (30, 82922) | 15886  (45,82402) | 14745 (32,75382) | 13440 (48,70642) | 11742 (23,69542) |

**Table S6**. The cFAR, cFAB and cACR for different methods on the simulated datasets of *H. sapiens* (CHM1).

|  | cFAR | cFAB | cARC | Sensitivity | Precision |
| --- | --- | --- | --- | --- | --- |
| smsMap | 99.84 | 99.78 | 99.99 | 99.7803 | 99.8602 |
| lordFAST | 99.64 | 99.64 | 99.99 | 99.6387 | 99.7318 |
| BLASR | 99.79 | 99.51 | 99.73 | 99.5061 | 99.7092 |
| BWA-MEM | 99.64 | 99.44 | 99.87 | 99.4441 | 99.6140 |
| GraphMap | 98.43 | 98.26 | 99.99 | 98.2560 | 98.4481 |
| minimap2 | 99.83 | 99.77 | 99.91 | 99.7744 | 99.8515 |
| NGMLR | 98.46 | 98.48 | 99.89 | 98.4807 | 99.1440 |
| rHAT | 99.71 | 99.47 | 99.96 | 99.4720 | 99.5220 |

This simulated of *H. sapiens* (CHM1) dataset includes 90,316 reads and 450.56 million bases with average error rate of 15%.

**Table S7**. Different SVs types and its breakpoints in the genome.

|  | SV type | Start position | End position | Length |
| --- | --- | --- | --- | --- |
| 1 | deletion | 247,888,169 | 247,894,336 | 6168 |
| 2 | insertion | 228,157,036 | 228,160,245 | 3210 |
| 3 | duplication | 77,010,679 | 77,011,077 | 399 |
| 4 | tandem | 118,387,369 | 118,387,460 | 92 |
| 5 | inversion | 2,553,995 | 2,567,688 | 13639 |
| 6 | tandem | 15,3256,662 | 153,256,746 | 84 |
| 7 | deletion | 229,676,353 | 229,685,091 | 8738 |
| 8 | inversion | 239,952,907 | 239,953,612 | 705 |
| 9 | deletion | 103,524,378 | 103,526,545 | 2167 |
| 10 | insertion | 143,734,439 | 143,760,271 | 25832 |
| 11 | insertion | 58,689,695 | 58,690,441 | 747 |
| 12 | duplication | 10,661,208 | 10,663,006 | 1799 |
| 13 | deletion | 62083833 | 62,095,479 | 11647 |
| 14 | inversion | 21,203,905 | 21,204,891 | 987 |
| 15 | deletion | 105,592,539 | 105,594,815 | 2277 |
| 16 | deletion | 22,563,407 | 22,563,807 | 401 |
| 17 | insertion | 160,984,674 | 160,984,805 | 123 |
| 18 | deletion | 62,123,776 | 62,123,827 | 52 |
| 19 | deletion | 54,821,892 | 54,822,204 | 313 |
| 20 | duplication | 93,875,914 | 93,876,215 | 302 |
| 21 | insertion | 192,223,111 | 192,223,231 | 121 |

**Table S8**. PacBio datasets website links

| Datasets | Website links |
| --- | --- |
| *E. coli* | <https://github.com/PacificBiosciences/DevNet/wiki/E.-coli-Bacterial-Assembly> |
| *C. elegans* | <https://github.com/PacificBiosciences/DevNet/wiki/C.-elegans-data-set> |
| *A. thaliana* | <https://github.com/PacificBiosciences/DevNet/wiki/Arabidopsis-P5C3> |
| *H. sapiens* | https://github.com/PacificBiosciences/DevNet/wiki/H_sapiens_54x_release |

**Table S9**. Reference genome website links

| Genome | Website links |
| --- | --- |
| *E. coli* | <https://s3.amazonaws.com/files.pacb.com/datasets/secondary-analysis/e-coli-k12-P6C4/polished_assembly.fastq.gz> |
| *C. elegans* | <http://datasets.pacb.com.s3.amazonaws.com/2014/c_elegans/40X/polished_assembly/polished_assembly.fasta.gz> |
| *A. thaliana* | <http://datasets.pacb.com.s3.amazonaws.com/2014/Arabidopsis/reads/polished_assembly.fasta> |
| *H. sapiens* | https://www.ncbi.nlm.nih.gov/assembly/GCF_000306695.2/ |

**Table S10**. Statistics of *E. coli*, *A. thaliana*, *C. elegans* and *H. sapiens* datasets

| Datasets | Reference genome length | Read number | Total bases | Average read length |
| --- | --- | --- | --- | --- |
| *E. coli* | 4,681,865 | 42,582 | | 106,765,908 | | --- | | 2,507 |
| *A. thaliana* | 130,857,836 | 22,837 | 187,687,752 | 8,218 |
| *C. elegans* | 104,169,699 | 44,396 | 510,357,920 | 11,495 |
| *H. sapiens* | 3,037,883,181 | 72,501 | 483,793,658 | 6,672 |

**Table S11**. FAR(%), FAB(%) and ACR(%) of eight methods on *E. coli* UTI89 dataset.

|  | smsMap | lordFAST | BLASR | BWA-MEM | GraphMap | minimap2 | NGLMR | rHAT |
| --- | --- | --- | --- | --- | --- | --- | --- | --- |
| FAR | 95.156 | 85.471 | 43.129 | 52.511 | 88.792 | 33.167 | 15.359 | 90.784 |
| FAB | 94.325 | 87.931 | 5.339 | 24.484 | 92.711 | 12.429 | 5.257 | 17.276 |
| ACR | 99.999 | 99.062 | 15.364 | 40.709 | 99.797 | 29.323 | 31.170 | 24.315 |

** E. coli* UTI89 dataset was generated by Oxford Nanopore Technologies MinION sequencers, and it is deposited in the European Nucleotide Archive under the accession code ERX987748.

**Table S12**. Agreement of different alignment methods for *A. thaliana* dataset

|  | smsMap | lordFAST | BLASR | BWA-MEM | GraphMap | minimap2 | NGMLR |
| --- | --- | --- | --- | --- | --- | --- | --- |
| smsMap | N/A | 80.77 | 78.03 | 77.57 | 86.40 | 78.90 | 74.55 |
| lordFAST | 78.75 | N/A | 75.26 | 80.01 | 86.63 | 81.15 | 75.58 |
| BLASR | 88.01 | 83.91 | N/A | 89.48 | 88.48 | 90.22 | 81.95 |
| BWA-MEM | 84.03 | 85.02 | 83.31 | N/A | 88.87 | 90.50 | 82.56 |
| GraphMap | 79.43 | 82.82 | 74.18 | 80.49 | N/A | 81.41 | 77.64 |
| minimap2 | 85.33 | 85.77 | 85.51 | 91.72 | 89.82 | N/A | 83.75 |
| NGLMR | 89.28 | 88.17 | 89.05 | 93.93 | 94.32 | 95.27 | N/A |

**Table S13**. Agreement of different alignment methods for *C. elegans* dataset

|  | smsMap | lordFAST | BLASR | BWA-MEM | GraphMap | minimap2 | NGMLR |
| --- | --- | --- | --- | --- | --- | --- | --- |
| smsMap | N/A | 84.49 | 85.40 | 82.37 | 92.47 | 85.45 | 79.75 |
| lordFAST | 82.90 | N/A | 82.46 | 84.85 | 94.76 | 88.01 | 81.91 |
| BLASR | 90.76 | 87.22 | N/A | 91.24 | 95.36 | 94.73 | 87.96 |
| BWA-MEM | 86.55 | 88.24 | 89.66 | N/A | 95.86 | 96.06 | 88.97 |
| GraphMap | 81.66 | 87.08 | 81.35 | 84.58 | N/A | 87.63 | 82.13 |
| minimap2 | 86.17 | 88.18 | 89.72 | 93.01 | 96.01 | N/A | 88.95 |
| NGLMR | 88.41 | 89.78 | 92.67 | 94.87 | 97.69 | 98.05 | N/A |

**Table S14**. Agreement of different alignment methods for *H. sapiens* dataset

|  | smsMap | lordFAST | BLASR | BWA-MEM | GraphMap* | minimap2 | NGMLR |
| --- | --- | --- | --- | --- | --- | --- | --- |
| smsMap | N/A | 79.35 | 78.80 | 75.80 | - | 79.05 | 72.82 |
| lordFAST | 80.18 | N/A | 74.43 | 78.99 | - | 82.71 | 75.56 |
| BLASR | 89.44 | 82.51 | N/A | 85.74 | - | 89.15 | 81.19 |
| BWA-MEM | 85.00 | 84.90 | 82.39 | N/A | - | 91.73 | 83.60 |
| GraphMap | - | - | - | - | - | - | - |
| minimap2 | 85.37 | 85.31 | 83.41 | 89.06 | - | N/A | 84.31 |
| NGLMR | 88.01 | 86.86 | 87.48 | 92.08 | - | 95.44 | N/A |

* GraphMap always appears core dumped information for *H. sapiens dataset*, it does not output the results.

**Table S15**. Aligned results of different alignment methods for one sequence. (see Figure S2 for detail alignments)

|  | Aligned positions (start-end) | Number of aligned bases | Aligned coverage (%) | Aligned identify (%) |
| --- | --- | --- | --- | --- |
| smsMap | 1-296 | 296 | 100 | 75.48 |
| lordFAST | - | - | - | - |
| BLASR | 94-289 | 196 | 66.22 | 88.46 |
| BWA-MEM | 93-296 | 204 | 68.92 | 87.96 |
| GraphMap | 93-296 | 206 | 69.59 | 87.96 |
| minimap2 | - | - | - | - |
| NGLMR | 93-296 | 206 | 69.59 | 87.93 |

minimap2 and lordFAST fail to align this sequence.

**Table S16**. The average and maximum of width coefficient for simulated datasets.

| Error rate (%) | 5 | 10 | 15 | 20 | 25 | 30 |
| --- | --- | --- | --- | --- | --- | --- |
| Ave. | 0.0018 | 0.0026 | 0.0033 | 0.0037 | 0.0038 | 0.0042 |
| Max. | 0.0059 | 0.0083 | 0.0104 | 0.0135 | 0.0111 | 0.0138 |
